# Hopes, concerns, satisfaction and regret in a precision medicine trial for childhood cancer: a mixed-methods study of parent and patient perspectives

**DOI:** 10.1038/s41416-023-02429-1

**Published:** 2023-09-19

**Authors:** Claire E. Wakefield, Kate Hetherington, Eden G. Robertson, Mark W. Donoghoe, Jacqueline D. Hunter, Janine Vetsch, Jonathan M. Marron, Katherine M. Tucker, Glenn M. Marshall, Alexander Broom, Michelle Haber, Vanessa Tyrrell, David Malkin, Loretta Lau, Marion K. Mateos, Tracey A. O’Brien, David S. Ziegler

**Affiliations:** 1 School of Clinical Medicine, UNSW Medicine and Health, UNSW Sydney, Sydney, NSW Australia; 2 Behavioural Sciences Unit, Kids Cancer Centre, Sydney Children’s Hospital, Sydney, NSW Australia; 3 Stats Central, Mark Wainwright Analytical Centre, UNSW Sydney, Sydney, NSW Australia; 4 Clinical Research Unit, UNSW Medicine and Health, UNSW Sydney, Sydney, NSW Australia; 5 Department of Obstetrics and Gynaecology, Royal Women’s Hospital, University of Melbourne, Melbourne, VIC Australia; 6 Department of Health, Institute of Applied Nursing Science, Eastern Switzerland University of Applied Sciences, St. Gallen, Switzerland; 7 Dana-Farber/Boston Children’s Cancer and Blood Disorders Center, Harvard Medical School, Boston, MA USA; 8 Center for Bioethics, Harvard Medical School, Boston, MA USA; 9 Department of Medical Oncology, Hereditary Cancer Centre, Prince of Wales Hospital, Randwick, NSW Australia; 10 Prince of Wales Clinical School, UNSW Sydney, Sydney, 2052 NSW Australia; 11 Kids Cancer Centre, Sydney Children’s Hospital, Sydney, NSW Australia; 12 Children’s Cancer Institute, UNSW Sydney, Sydney, NSW Australia; 13 Sydney Centre for Healthy Societies, School of Social and Political Sciences, The University of Sydney, Sydney, NSW Australia; 14 Division of Hematology/Oncology, The Hospital for Sick Children, Toronto, ON Canada; 15 Departments of Pediatrics and Medical Biophysics, University of Toronto, Toronto, ON Canada

**Keywords:** Cancer genetics, Cancer genomics, Paediatric cancer, Paediatric research, Human behaviour

## Abstract

**Background:**

Paediatric precision oncology aims to match therapeutic agents to driver gene targets. We investigated whether parents and patients regret participation in precision medicine trials, particularly when their hopes are unfulfilled.

**Methods:**

Parents and adolescent patients completed questionnaires at trial enrolment (T0) and after receiving results (T1). Parents opted-in to an interview at T1. Bereaved parents completed a questionnaire 6-months post-bereavement (T1B). We analysed quantitative data with R and qualitative data thematically with NVivo, before integrating all data for interpretation.

**Results:**

182 parents and 23 patients completed T0; 108/182 parents and 8/23 patients completed T1; 27/98 bereaved parents completed T1B; and 45/108 parents were interviewed. At enrolment, participants held concurrent hopes that precision medicine would benefit future children and their child. Participants expressed concern regarding wait-times for receipt of results. Most participants found the trial beneficial and not burdensome, including bereaved parents. Participants reported high trial satisfaction (median scores: parents: 93/100; patients: 80/100). Participants expressed few regrets (parent median scores: parents: 10/100; bereaved parents: 15/100; patient regret: 2/8 expressed minimal regret).

**Conclusions:**

Even when trial outcomes did not match their hopes, parents and patients rarely regretted participating in a childhood cancer precision medicine trial. These data are critical for integrating participants’ views into future precision medicine delivery.

## Background

Precision medicine (PM) represents a paradigm shift in oncology. It involves collating information collected through tumour profiling, including genomics and drug screening, to provide additional diagnostic information and to allow the identification of personalised treatments [1]. Given the anticipated benefits of PM, clinical trials are testing the feasibility and clinical utility of its application in childhood cancer worldwide [1, 2]. While PM holds promise, most patients will not receive a precision-guided treatment that leads to clear clinical benefit [1, 2].

It is critical to understand the experiences of those who receive any new medical treatment, as patient engagement is essential for successful widescale implementation, especially now that patients increasingly expect to be involved in medical decision-making [3]. Early data suggest that patients harbour multifaceted hopes and concerns regarding PM [3–7]. Many approach PM with ‘high hopes’, despite a minority experiencing clinical benefit [5, 6]. Hopes include altruistic aspirations for future patients and a desire to support the progress of science, whilst simultaneously hoping to receive more information about their tumour, additional treatment options, and ultimately, a greater chance of cure [3, 6, 7].

Patients’ hopes are often numerous, leading to clinician and ethical review board apprehension about potentially instilling unrealistic expectations or ‘false hopes’ [8]. PM trials may not identify any therapeutic options for patients, or they may identify potentially beneficial therapies that patients cannot access [9]. From the limited available literature, patients’ concerns about PM appear less prevalent than clinicians, although they report worrying about wait-times for results, receiving ‘bad news’, and potential privacy/insurance impacts [3, 6]. Parents’ deeply-held hope for a cure, their desire to be a ‘good parent’ and their eagerness to ‘try everything’ may also impact their capacity to fully understand the implications of their child participating in PM [3, 10, 11].

As many PM trials now straddle the line between research and clinical care [4], it is necessary to understand any community concerns to maximise participant engagement and ensure that the promise of PM can be fully realised. Yet, a recent synthesis of 92 studies [12] revealed that no large-scale, prospective, whole-family studies are available to guide the implementation of future PM trials in childhood cancer. Studies that do not follow participants over time, and those that exclude the views of bereaved parents, likely limit representation of negative or suboptimal experiences. In fact, in the poor-prognosis setting where bereavement is the most likely outcome, the views of bereaved parents are particularly important. Few studies have also included the ‘voice of the child’, despite the United Nations Convention on the Rights of the Child and the US Food and Drug Administration ratifying the importance of including of child/adolescent perspectives [13]. To fill these gaps, we addressed these questions:

1. What are parents’ and adolescent patients’ hopes and concerns when enroling in a childhood cancer PM trial?
2. Is participating in a PM trial beneficial or burdensome for parents and patients, and do their perceptions change over time?
3. How satisfied are participants, does satisfaction change over time, and how are changes in satisfaction associated with receiving a treatment recommendation or change in cancer treatment through a PM trial?
4. Do parents and patients experience any regrets about having participated?

## Methods

*‘PRecISion Medicine for Children with Cancer’* (PRISM) was an Australian PM trial for children with cancer and an expected survival rate of <30% open at all eight children’s hospitals in Australia [1]. PRISM was delivered through the ZERO Childhood Cancer Program (NCT03336931). PRISM included a comprehensive analysis of the child’s tumour cells, genetic/genomic testing of tumour and non-tumour cells, and biological models such as drug testing of tumour cells in-vitro and in patient-derived xenografts. A multidisciplinary tumour board assessed the data derived from these tests and where possible, provided treatment recommendations. The recommendations were then shared with the child’s oncologist, who could choose to refine the child’s treatment in consultation with the family. The timeframe from trial enrolment to receipt of results/recommendations was 8–12 weeks. The ‘PRISM-Impact’ study ran alongside PRISM and aimed to understand better parents’/patients’ experiences of PM (HREC/17/HNE/29).

### Population

PRISM patients were eligible if they were ≤21 years and diagnosed with any malignancy with a low chance of cure (<30%), either at diagnosis or relapse. PRISM patients aged 12–17, and all parents/caregivers, were eligible for PRISM-Impact if they had sufficient English language skills to participate and did not present with severe cognitive/physical/mental health concerns as assessed by the child’s treating team. English language skills were also determined by the child’s treating team, who used their clinical judgement to assess language skills and, where appropriate, asked families whether they felt comfortable reading and understanding written and conversational English. Those who did not were deemed ineligible for PRISM-Impact but were still eligible for the main PRISM clinical trial. Parents whose child died during PRISM remained eligible, however we did not contact parents until after six-months post-bereavement (aligning with guidance) [14].

### Recruitment

Participants opted-in to PRISM-Impact through the PRISM consent form. We telephoned parents after they opted-in to assess their questionnaire preference (online via Qualtrics^TM^/paper) and confirm their interest in the optional telephone interview at T1. If participants indicated significant distress, determined as a rating of 8 or above out of 10 on the distress item of the Emotion Thermometers scale [15], participants were offered a call from our study psychologist to provide referral options.

### Data collection and measures

We developed and pilot-tested the questionnaires/interviews with a multidisciplinary expert panel.

#### Questionnaires (Supplementary Tables 1, 2)

We sent participants a baseline questionnaire shortly after trial enrolment (Time 0, T0) and a second questionnaire 2–4 weeks after the return of the child’s results/recommendations (T1). We sent bereaved parents a questionnaire tailored to their situation (T1B). We followed up on missing questionnaires with up to three telephone calls.

We collated clinical information (e.g. cancer type) from hospital electronic medical records. PRISM-Impact questionnaires included validated and purpose-developed items based on available literature and expert opinion on participants’ hopes and concerns, perceived benefits/burdens of trial participation, satisfaction with participating, whether participants would recommend the trial to others, and any regrets [6, 8, 16–18]. Questionnaires allowed free-text responses for additional information. Patients’ questionnaires assessed the same domains as parents’, with fewer/simpler questions to minimise burden.

#### Interviews (T1)

Augmenting the questionnaire, semi-structured telephone interviews encouraged parents to discuss their experiences with PRISM, probing for detail on the above topics (Supplementary Table 3). Three researchers with no prior relationship with participants conducted the interviews, which were audio-recorded and transcribed verbatim.

### Data analysis

We adopted a quantitative-dominant mixed-methods approach [19], focussing on quantitative data analysis, then examining the qualitative data, before using side-by-side joint displays to integrate all findings [20]. We used the qualitative data to enhance our understanding of the quantitative data, extracting illustrative quotes [20].

#### Quantitative analyses

We analysed quantitative data using SPSS(v24·0)/R(v4·1·2) [21, 22]. To investigate changing perceptions over time we excluded data from families who were not due to participate in T1 at the time of analysis. To assess changes between T0 and T1/T1B, we fit mixed-effects regression models that included random-effects for participants nested within families, allowing us to account for the correlation between individual parent’s responses over time, and between parents of the same patient. We performed Wald tests to assess the statistical significance of the coefficient associated with time. To analyse changes in willingness to recommend PRISM, we performed a McNemar–Bowker test of symmetry on the 3 × 3 table of paired nominal responses, followed by post-hoc McNemar tests on the 2 × 2 subtables, with a Holm–Bonferroni adjustment for multiple comparisons. We used a mixed-effects Tweedie regression model to estimate the association between parent satisfaction with participation at T1 and both whether they received any treatment recommendations, and whether the PRM report led to a change in treatment, adjusting for parent satisfaction with participation at T0.

#### Qualitative analyses

We conducted a directed content analysis of participants’ responses to free-text questionnaire items. We used thematic analysis with an inductive approach [23] for interview data. Two coders (JH/CW) independently coded all qualitative data, and then discussed their findings to reach consensus. We then aligned these themes with the quantitative dataset to create a cohesive overview.

### Role of the funding source

The funders of this study played no role in study design, data collection/analysis/interpretation, or writing of the paper.

## Results

### Participants

Table 1 summarises demographic and clinical characteristics of participating patients and children of participating parents. Supplementary Table 4 summarises the demographics of participating parents. Supplementary Fig. 1 (Consort Diagram) summarises enrolment, response rates, and participation in each trial phase. 108 parents from 93 families completed both T0 and T1. 27/98 parents whose child died completed T1B (28%). 23 adolescent patients completed T0, while 8 completed both T0 and T1. Of 98 parents with complete satisfaction data, 32 did not receive a treatment recommendation for their child, 40 received a recommendation but their child’s treatment was not changed, and 26 parents received a recommendation that led to a treatment change for their child.

**Table 1:** Demographics of participating patients and children of participating parents in PRISM-IMPACT.

	Characteristics of children whose parents completed questionnaires (*N* = 144)	Characteristics of children whose parents completed an interview (*N* = 43)	Characteristics of patients who completed questionnaires (*N* = 23)
Age of child at enrolment in PRISM, years			
Mean (SD)	9.1 (5.5)	9.0 (5.7)	14.9 (2.1)
Range	0–18^a^	1–17	12–18^a^
Age of child at diagnosis, years			
Mean (SD)	8.0 (5.5)	7.9 (5.6)	13.0 (2.8)
Range	0–17	0–17	7–17
Sex, no. (%)			
Female	71 (49.3%)	20 (46.5%)	14 (60.9%)
Male	73 (50.7%)	23 (53.5%)	9 (39.1%)
Diagnosis, no. (%)			
Central nervous system tumour	57 (28.5%)	16 (37.2%)	5 (21.7%)
Sarcoma	41 (39.6%)	13 (30.2%)	12 (52.2%)
Leukaemia/Lymphoma	21 (14.6%)	4 (9.3%)	4 (17.4%)
Neuroblastoma	14 (9.7%)	4 (9.3%)	0 (0%)
Other^b^	11 (7.6%)	6 (14.0%)	2 (8.7%)
Child relapse prior to PRISM enrolment, no. (%)			
Yes	81 (56.2%)	19 (44.2%)	12 (52.2%)
No	63 (43.8%)	24 (55.8%)	11 (57.8%)

*SD* standard deviation, *PRISM* PRecISion Medicine for Children with Cancer, *PRISM-Impact* the psychosocial sub-study running alongside the PRISM study, *CNS* central nervous system.

^a^The age restriction was based on the child’s age at the date of consenting to PRISM, while the age summarised in the table is that reported by the parent at baseline. Hence it was possible to include children aged 18 at baseline, if they had their birthday in between PRISM consent and baseline.

^b^Participants affected by rare cancers are presented as ‘other’ throughout the manuscript to reduce the chance that they could be identified by their rare diagnosis.

#### Hopes (Fig. 1a and Table 2)

Parents held multiple concurrent hopes when they enroled their child in PRISM, hoping that PRISM would find cures for future cancer patients (98% of parents) and also provide information about their child’s cancer (89%). Most parents hoped that PRISM would provide their child with more treatment options (82%) and increase their child’s chance of cure (78%). Qualitative data confirmed parents’ intertwined hopes: ‘*Providing hope for treatment for my son. Hope that the research will improve outcomes for other children. Hope that the research will provide information which will be useful to researchers/society*’ (mother, 44 years of age). Parents hoped that PRISM would identify new treatment options their clinicians had not considered and would provide reassurance that they had tried all avenues to save their child, that they had acted as ‘good parents’, and that they had not failed their child.

**Fig. 1:**
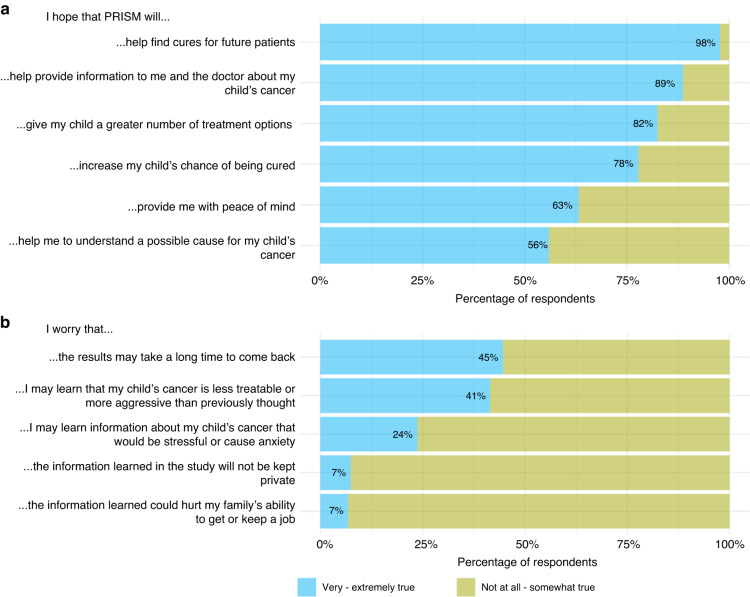
Parents’ hopes and concerns at enrolment in the PRISM trial. **a** Hopes. **b** Concerns. Participants indicated their level of agreement with 11 separate questions, comprising the 6 hopes and 5 concerns listed in this figure.

**Table 2:** Illustrative quotations regarding participants’ hopes and concerns when enroling in PRISM.

Parents	Patients
Hopes	
Help find cures for future patients through research	
I believe that everyone should participate in medical studies wherever possible. We are currently receiving treatment that would not have been possible without thorough research, and it is nice to pay it forward. Even if it doesn’t help our daughter, hopefully it will help others in the future. (mother, 35, child with leukaemia) Given us hope, however small, if not for our family, then for others. (father, 31, child with CNS tumour) Knowing it’s going to help other people. (father, 44, child with leukaemia) Hoping this will help…other kids in the future. (father, 46, child with CNS tumour) Knowing it may help future cancer patients is comforting. (mother, 40, child with neuroblastoma) If being part of the PRISM study can help anyone with cancer, I am all for it. (mother, 49, child with CNS tumour) I’d like to think it will be helpful to my daughter and others (father, 36 child with leukaemia)	Good idea, may not help me, but probably will help somebody else. (patient, 12, other tumour) Knowing that they will hopefully find something that can be helpful to future patients and doctors/scientist. (patient, 7, CNS tumour) It’s given me the chance to possibly help myself and others. (patient, 15, sarcoma) The chance to not only have a cure for myself but for others as well. (patient, 13, sarcoma) It offers hope to me and future patients to be cured. (patient, 13, sarcoma) Knowing that being part of PRISM could increase the likelihood of developing cures for my type of cancer in the future. (patient, 17, sarcoma) Because in a way I know I’m helping with research (patient, 14, sarcoma) I’ll be helping cancer research and maybe help find a cure for cancers (patient, 16, leukaemia) I’d have to say it has made me feel happy because in today’s day and age we are able to do so much more with information and discoveries. (patient, 14, sarcoma) Knowing there are people out there using leftover samples to try to get a cure. (patient, 12, sarcoma)
Provide more information about the child’s cancer	
It is one of the only options available to my child. It will give my oncologist a depth of information they won’t receive elsewhere. (father, 39, child with neuroblastoma) To help with a diagnosis and learn more about his tumour which is currently undiagnosed. (mother, 42, child with other tumour) Opportunity to gain further information about child’s condition. (mother, 47, child with CNS tumour) Getting information on our child’s disease. (father, 41, child with other tumour) More information about daughter’s tumour. (mother, 35, child with CNS tumour) It can lead to answers concerning my child’s cancer. (father, 55, child with sarcoma) We wanted to ensure [patient]‘s biopsy could provide us with additional data. (father, 40, child with CNS tumour)	To know through this study an answer or information could be found about the tumour. (patient, 16, sarcoma) Happy that they could find out some new research about what I have. (patient, 16, sarcoma) I thought it might find out some reasons why my cancer keeps coming back. (patient, 12, sarcoma) Knowing that people will be trying to understand my tumour and what makes it tick. (patient, 17, CNS tumour)
Provide tailored treatment options for the child, increase their chance of cure and reduce side effects	
Looking for alternative treatment options for my child who has poor outcome and high-risk cancer and relapse from previous treatment. (mother, 42, child with lymphoma) To hopefully discover a possibly beneficial treatment for our son’s cancer as we have exhausted all possible treatment options to date. Due to the rarity of our son’s cancer, there is little research regarding treatment options. (mother, 44, child with other tumour) I think the potential to find a treatment that is specifically tailored to fight my son’s cancer is what impressed me. (mother, 42, child with neuroblastoma) Talking on behalf of the patient … a baby. We’re hoping this program will increase a longer life for her to enjoy more things without too many obstacles or restrictions. (mother, 29, child with other tumour) Gives us hope for new treatment. (father, 35, child with CNS tumour) It does give me hope that the information the study gets from my child’s biopsy, may tailor a better treatment plan, thus be more effective and hopefully less side effects. (mother, 56, child with CNS tumour) I trust our doctor who is one of the researchers and want to find a cure. (mother, 50, child with other tumour) We weren’t given very positive information from the doctors about the survival of children with Ewing’s sarcoma, so it was very important to us that we look at all avenues to ensure that our daughter survives this. (mother, 47, child with sarcoma)	I just want to find a cure for my cancer. (patient, 14, sarcoma) I … hope there may be a treatment found that could help. (patient, 15, lymphoma) I was also very happy because the thought that there will be more treatment options available. (patient, 17, sarcoma) I hope you are able to stop my cancer coming back again. Two times is already enough. (patient, 12, other tumour) Going to find better treatment option. (patient, 17, CNS tumour) That they might find a way to get rid of the cancer. (patient, 12, sarcoma) Maybe you will find a better chemo drug to keep it away and not ever come back. (patient, 12, other tumour) To find cure (patient, 17 CNS tumour) I do have a backup or fall-back plan if conventional treatment doesn’t work. (patient, 15, lymphoma)
Provide peace of mind	
Just to know there could be other treatment plans on the way to help my daughter and that it could help lots of children in the future it puts me at ease knowing that. (father, 36, child with other tumour) I’ve done everything I could for my kid. Maybe find some useful information. Help scientists or other patients. (father, 43, child with CNS tumour) It’s good to know very clever people are trying. (a little) (father, 42, child with CNS tumour) It’s slightly reassuring feeling that there are people working behind the scenes for my child. (father, 39, child with CNS tumour) We are in the hands of our oncologist and the health care team as to any course of action for our sick child and we must trust that they will give their best efforts.’ (father, 43, child with CNS tumour)	Gives a bit of reassurance to being cured of cancer eventually (patient, 15, lymphoma) Because it helps my parents feel better with having a widespread range of medical opinions (patient, 12, CNS tumour) People are putting in heaps of effort to help me and people with cancer in the future. (patient, 13, CNS tumour) That something is being done to help me and other kids in the world (patient, 12, CNS tumour)
Understand the cause of the child’s cancer	
To understand possible causes of his neuroblastoma. (father, 40, child with neuroblastoma) I am really interested to see what is driving her tumour. (father, 40, CNS tumour)	
Concerns	
Parents	Patients
The time delay: cancer may progress while waiting for results	
Waiting is the hardest part. (mother, 42, child with CNS tumour) I understand PRISM is new, but the estimated time of when it’s possible to get results is long…12 weeks might not seem long but when told my child’s only chance of stability or improvement is chemotherapy and not surgery or radiation, then 12 weeks feels like it’s too long based on how serious and precious time is. The window of opportunity to treat something like this becomes smaller as tumours become bigger, if chemo isn’t working. (mother, 29, child with other tumour)	It made me feel worried that it takes a long time and the possibility of the cancer spreading. (patient, 13, sarcoma) That you need to use new drugs on me this time for chemo. If you find something better, I have already started a different chemo. (patient, 12, other tumour: unknown) If any new treatment comes up, will you change any of my chemo drugs that I am using as I think that PRISM study will take 3 months for any information to be gathered and I will have nearly finished my chemo treatment. (patient, 12, other tumour) When will I get my results? (patient, 14, sarcoma)
Information learned may be stressful or cause anxiety	
It’s about the ‘timing’—it is quite a lot to take in given the nature of our future. (mother, 49, child with neuroblastoma) Having to think about my child’s prospects. (father, age unknown, child with leukaemia) Reading the patient information sheet about high-risk cancers and low cure rate is not something a parent wants to hear, and is further anxiety provoking. (mother, 39, child with CNS tumour) Nervous about results. (mother, 35, child with CNS tumour)	
Uncertainty about the trial and the possible next steps for the child, unanswered questions	
What made you worried? ‘sometimes I am unsure to what we are agreeing to’. (mother, 45, child with sarcoma) The PRISM study allows no ability for the patient or family talk to principal researchers prior to consenting or surgery. No understanding that the families time is very precious.’ (father, 55, child with CNS tumour).	Being aware of what it going on constantly but not fully understanding can make me feel stressed. (patient, 17, CNS tumour) Because I don’t know what you guys will do to me. (patient, 12, other tumour What could happen to me? (patient, 16, lymphoma) What operations and procedures I will have to go through? (12, other tumour) What made you worried? Everything about the study, because I’m confused by it (patient 17, CNS tumour) What will you guys do? What procedures are available? What will happen to me if my tumour [keeps] growing? (patient, 12, other tumour)

All adolescent patients hoped that PRISM would find cures for future patients (100%), and almost all hoped to increase their own chance of cure (91%). These intertwined hopes were confirmed in patients’ qualitative responses: ‘*That you guys could have a chance to stop my tumours from growing and save other kids while you’re at it*.’ (patient, 12). Patients shared that they hoped PRISM would reduce their chance of cancer recurrence, providing a ‘*fall-back plan’* (patient, 15) if their current treatment failed. Patients shared that PRISM provided peace of mind for themselves and others, particularly their parents: ‘*it helps my parents feel better’* (patient, 12).

#### Concerns (Fig. 1b and Table 2)

Despite often describing positive views regarding the trial, parents also expressed concerns, most commonly regarding the timeline to receive results (45%) and fear that they might receive disappointing news about the aggressiveness of their child’s cancer (41%). Parents described deeply emotional responses to the wait-time, emphasising the urgency of accessing treatment, the shrinking window of time to achieve a cure, and a perceived race to intervene before their child’s cancer progressed. ‘*12-weeks feels too long based on how serious and precious time is. The window of opportunity to treat something like this becomes smaller as tumours become bigger*.’ (mother, 29).

Like parents, patients felt concerned about the length of time to receive results (39%), while others worried about PRISM causing extra stress for their family (17%) or themselves (17%). Patients’ qualitative responses also revealed concerns about commencing a (suboptimal) treatment while awaiting results. ‘*If you find something better, I have already started a different chemo*.’ (patient, 12).

#### Perceived benefit and burden

**Fig. 2:**
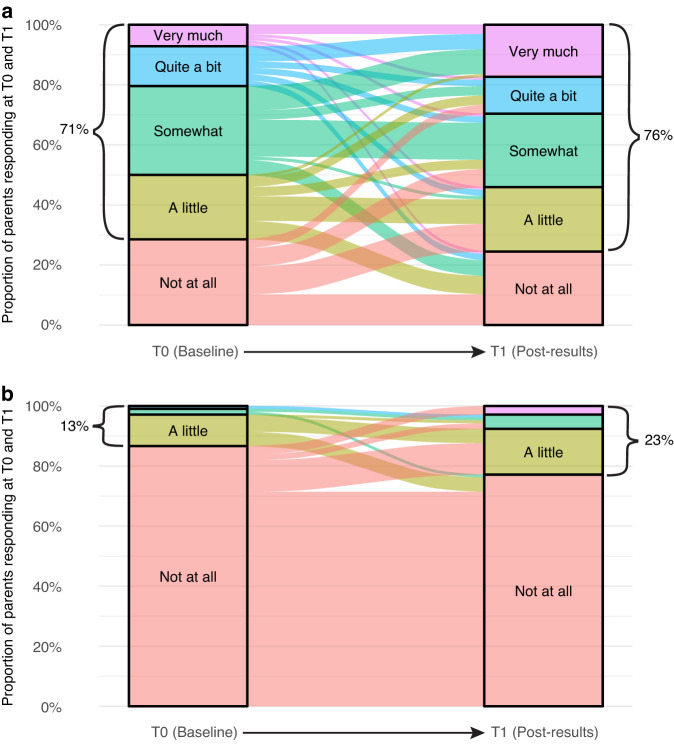
Parents’ perceptions of benefit and burden from participating in PRISM (only including those who responded at T0 and T1). **a** Perceptions of benefit. **b** Perceptions of burden. The alluvial plots in this figure visually represent the proportion of participants’ responses to the benefit and burden questions at Time 0 and Time 1, as well as any changes in participants’ individual responses over time. To assess change in participants’ responses over time, we used the clmm function from the ordinal R package to fit a cumulative logistic regression model for the ordinal outcomes relating to perceived benefit and burden.

At both timepoints, most parents rated participating in PRISM as at least ‘a little’ beneficial (T0:68%; T1:74%) and ‘not at all’ burdensome (T0:84%; T1:76%; Fig. 2a, b). Over time, parents became more likely to report both a higher level of benefit from the trial (OR1.78; 95%CI:1.08–2.93; *p* = 0.024*)* and a higher level of burden (OR 3.57; 95% CI:1.36–9.38; *p* = 0.010). Parents’ qualitative feedback revealed that for most, the benefits outweighed any burdens. ‘*Oh, hugely grateful. Hugely, hugely grateful. One of the best decisions we made’* (mother, 35).

Most bereaved parents continued to report benefits (54%) and that trial participation was ‘not at all’ burdensome (70%). Bereaved parents valued being able to ‘*save another family’s pain’* (mother, 42). They appreciated any extra time with their child, improved quality of life, and prolongation of hope afforded by the trial: ‘*Bought my daughter a few more months of quality life…it was something that money couldn’t buy*’ (father, 55); ‘*Ultimately PRISM couldn’t save my son’s life, but it gave us hope that we could find a way and that was far better than just going home to make memories*’ (mother, 39). Some bereaved parents shared administrative burdens of participating: ‘*The paperwork was a bit of a pain…while we had more important things to worry about*’ (father, 55). Others shared their distress at receiving no recommendations or ineffective treatments. ‘*Offered hope, but then failed to deliver on treatment’* (mother, 56).

Most adolescent patients reported at least ‘a little’ benefit from participating at T0 (65%), with half reporting at least some benefit at T1 (50%). Most patients reported that the trial was ‘not at all’ burdensome (T0:87%; T1:75%). Patients shared that they found it beneficial ‘*to hear there are others like me’* (patient, 12), describing comfort that other young people were also participating in the trial. Some patients perceived burdens on their parents with limited benefit: ‘*My parents were spending too much time on (PRISM); such as sending samples/information, when PRISM won’t give back any results*’ (patient, 12).

#### Satisfaction

**Fig. 3:**
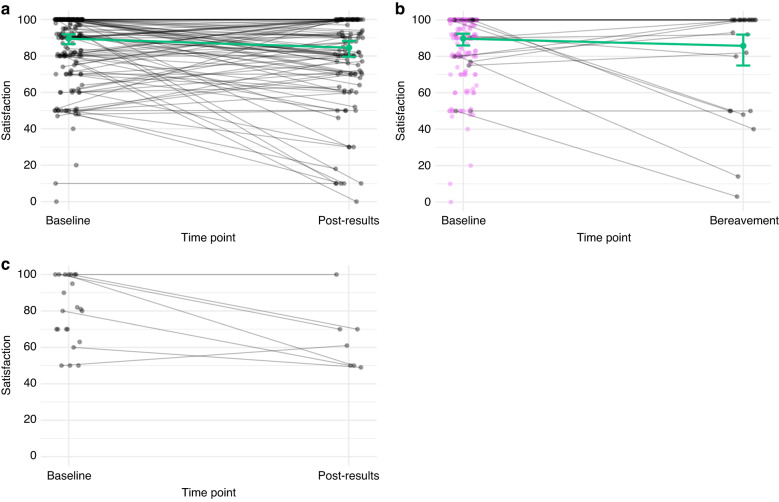
Parents’, bereaved parents, and patients’ satisfaction with PRISM participation, with fitted means. **a** Non-bereaved parents. **b** Bereaved parents. **c** Patients. We used the glmmTMB R package to fit a Tweedie regression model to the deficit from 100 in satisfaction, checking simulated probability inverse transform residuals to ensure a reasonable fit.

**Fig. 4:**
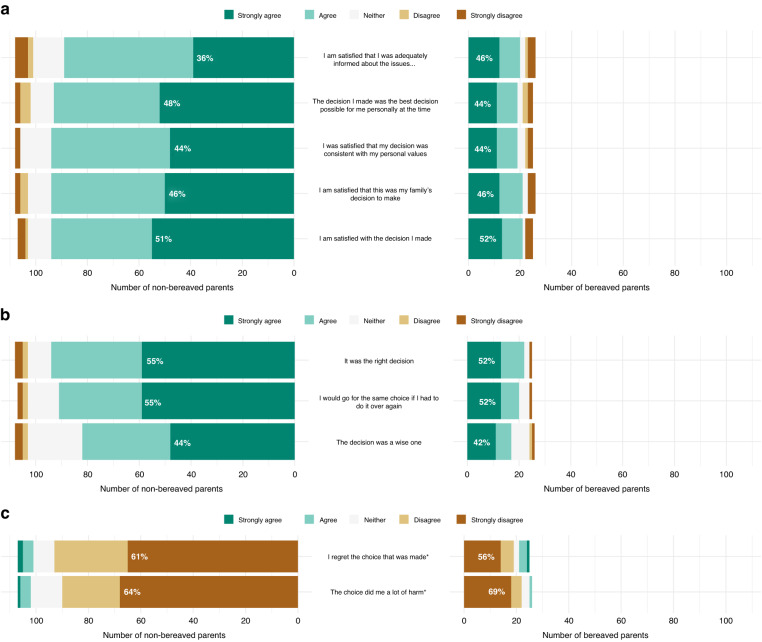
Non-bereaved parents’ and bereaved parents’ responses to five items in the Satisfaction with Decision Scale (T1 and T1B) and five items in the Decision Regret Scale (T1 and T1B). **a** Satisfaction with Decision Scale. **b** and **c** Decision Regret Scale. We used the mgcv R package to fit a generalised additive mixed-model for parents’ regret scores, modelled as coming from a Tweedie distribution. The model included a random effect for family and pre-specified fixed effects of interest: parent sex, parent education level, rurality, child’s cancer type, child’s age at enrolment, and whether the parent was bereaved prior to completing T1. We estimated ~95% confidence intervals for fitted-coefficients using asymptotic normality, and used Wald tests to assess statistical significance.

At T0, parents’ satisfaction was high (median: 93/100), with 42% of parents giving the maximum response of 100/100 (Figs. 3 and 4a). At T1, parents’ median satisfaction rating was 85/100, with 33% scoring 100. Most parents indicated that that their decision was the best option at the time and that it was consistent with their values. There was a small but significant reduction in satisfaction ratings between T0 and T1 (T0:90; 95%CI = 87,92; T1:85; 95%CI = 80,88; *p* = 0.004). Notably, satisfaction was high for parents who became bereaved (T0:90/100, 95%CI = 86,92; T1B: 86/100, 95%CI = 75,92; *p* = 0.26). When asked to elaborate on any dissatisfaction with PRISM, some parents commented on a lack of communication from PRISM, revealing that they expected more timely information about the trial’s progress and their child’s results. ‘*We have no communications. All that was done was sending a sample to Sydney to PRISM to be tested. Nothing else was introduced/actioned*’ (father, 48).

Parents who received a recommendation without a change in cancer treatment had an estimated post-results satisfaction deficit (shortfall from the maximum score of 100) that was 0.79 times (95%CI = 0.48,1.31; *p* = 0.358) that of parents whose child did not receive a treatment recommendation, after adjusting for baseline satisfaction. Those who received a recommendation and had a change in treatment had an estimated post-results satisfaction deficit that was 0.54 times (95%CI = 0.26,1.03; *p* = 0.066) that of those who did not receive any recommendations, after adjusting for baseline satisfaction. Overall, the association between T1 satisfaction and receiving a recommendation and/or a change in treatment was not statistically significant (*p* = 0.179).

Patients reported high satisfaction at T0 (median:80/100). At T1, patients’ median satisfaction rating was 61/100.

Most parents reported that they would recommend PRISM to other parents at all timepoints (T0:76%; T1:87%; T1B:78%). The distribution of parents’ responses changed over time, with the largest change being ‘unsure’ parents at T0 who shifted to stating they would recommend PRISM at T1 (*p* = 0.002). At T0, 17/23 of patients reported that they would recommend PRISM to other patients, while 4/8 patients were willing to recommend the trial at T1.

Qualitatively, parents were enthusiastic about recommending the trial to others. ‘*Yes. Because the more we know about our children’s cancers, the better opportunities there will be to find more effective treatments*’ (mother, 36). Despite overall enthusiasm, some parents expressed caution regarding fostering unrealistic hopes, acknowledging the importance of individual choice: ‘*I’d be careful not to give them false hope, because we know that it doesn’t always end in results for your child*’ (father, 31).

#### Regret (Fig. 4b, c)

Parents’ T1 regret scores were low (median:10/100), with most parents indicating that the decision did not cause them harm and that they would make the same decision again. Bereaved parents’ scores were also low (median:15/100). Using a regression model, we did not observe evidence of an association between regret and parent sex, parent education level, rurality, the child’s cancer diagnosis, child’s age, or parents becoming bereaved. No patients indicated that it was ‘very’ or ‘extremely’ true that they regretted being part of this study. Most patients (*n* = 6/8) indicated that it was ‘not at all true’ that they regretted participation, with the remainder indicating that it was ‘a little true’ (*n* = 1) or ‘somewhat true’ (*n* = 1) that they regretted being part of the study.

## Discussion

This national prospective study explored parents’ and adolescent patients’ experiences of participating in a PM trial for childhood cancer, uniquely giving voice to mothers and fathers, adolescent patients, and bereaved parents. Our data are novel in having been collected shortly after trial enrolment and after receipt of PM results/recommendations, adding to early studies which asked participants to retrospectively reflect on their hopes only after results had been shared [6]. The results show a high level of satisfaction at both time points, low regret, and perceived benefit for most participants, including bereaved parents.

Parents/patients experienced multiple coexisting hopes and concerns upon trial enrolment, hoping the trial would benefit future children and their child/themselves, as well as worrying about cancer progression during the time taken to receive results. Our study did not assess whether participants’ hopes were ‘realistic.’ This would have been challenging given the heterogenous and rare cancer types included, and the fact that the PRISM trial involved identifying potential experimental treatments with limited current evidence of medical benefit. Some families do not fully understand the goals of clinical trials in paediatric oncology [5, 24], with a few having unrealistic hopes for direct medical benefit [25]. PM trials add a layer of complexity for participants, as they are not directly therapeutic. Rather, they provide diagnostic analyses with therapeutic implications, potentially influencing participants’ understanding and their resulting hopes regarding their participation.

As others have recognised [3, 26], hope is a complex and nuanced phenomenon influenced not only by an individual’s disposition but also by their understanding of likely outcomes. Patients in this study had a low chance of cure (<30%), however hope can persist and assist with coping alongside an awareness of a patient’s poor prognosis and can be multi-factorial, such that coexisting hopes can be challenging to disentangle [27]. Our qualitative data revealed hopes that have not been well-documented previously, including hopes to achieve peace of mind from having ‘tried everything’ and for parents, having been the best parent for their child. Adolescent patients’ focus on others (their parents and future patients), rather than themselves, was potent, revealing a relatively mature awareness of the relational elements of cancer [28] and the impacts of caregiving on their parents [7].

It was unsurprising that participants worried about waiting for their results/recommendations. Participants described fears that cancer would progress or evolve while waiting, making recommendations less useful over time. Cancer patients/caregivers are acutely aware of the salience of time, the urgency of commencing treatment, and the diminishing possibility of achieving cure as time passes [29]. The impact of ‘waiting’ on parents and young patients is underexplored [29], especially in the context of high-risk disease. Our data reinforce the value of continued scientific innovation to enable more rapid result derivation and shorten wait-times, along with the development of processes for communicating results to families as quickly as possible.

Our data regarding perceived benefits/burdens of participation were largely reassuring. Echoing earlier smaller-scale single-timepoint studies [3, 6, 7], perceived benefits typically outweighed burdens. While clinicians can be hesitant to add burden to patients with a poor prognosis, our participants rarely reported significant burdens. This is important, as clinicians may find it difficult to offer enrolment on trials unlikely to provide direct benefit to patients with a poor prognosis. Our data suggest that these patients/families may still derive benefit, possibly through their altruistic contribution toward others. Perhaps the greatest burden shared by our participants was lost time: time spent participating in the trial rather than spending time with the child. This was observed particularly by bereaved parents reflecting on their last days spent with their child. This was, however, possibly counterbalanced by other families who perceived that the trial had afforded them extra time with their child.

Participants reported high satisfaction, although some parents were frustrated by limited communication about the trial and their child’s results, and others were disappointed that the trial did not yield actionable results. It is possible that as hopes faded, so too did satisfaction. Yet, most participants remained willing to recommend the trial to others, suggesting a collective view of encouraging others to ‘buy-in’ into the trial, despite their individual outcomes. In fact, unsure parents became *more* likely to recommend the trial over time, rather than *less*. Whilst we were not able to explore reasons for this in this study, parents may have become more likely to recommend the trial due to positive impacts experienced, such as providing parents with peace of mind that they had exhausted all options to save their child. It is also possible that having gone through the process, parents felt more confident that they understood the possible impacts of the study, and therefore were more comfortable recommending to others. Satisfaction may have declined with ‘waiting’ or with news that nothing further could be done to save the child, although our bereaved parent data suggest that satisfaction was not solely driven by the child’s clinical outcome, at least at six-months post-bereavement. Future studies should explore the relationships between satisfaction and hope, clinical outcomes (including whether the child receives a treatment recommendation and/or change in treatment), and wait-time. Improving communication with families will be important for future PM trials, to ensure that family expectations regarding receiving results are met. Communication that not only provides the patient’s results, but also reinforces the most likely outcomes for the trial (for example, benefits for future children) while also acknowledging the tensions families experience in hoping, and in waiting, could be particularly helpful.

Powerfully, despite some concerns, few parents or patients expressed regrets regarding trial participation, even if the parent became bereaved. In fact, parents’ regret scores appeared comparable or lower than in other studies using the same regret measure in clinical trials for lower-risk cancers [30]. This is important, given the close links between regret and poorer mental health in patients [31].

A key strength of our large-scale study was its longitudinal design, allowing examination of changes before and after receiving trial results/recommendations. Participant engagement was high, yielding the largest available dataset addressing this topic to date. We also uniquely included bereaved parents, providing them with an opportunity to contribute to research and make meaning of their child’s death, without adding significant burden. Although the sample was small and attrition high, inclusion of adolescents added nuance to the study, revealed their eagerness to engage in PM, and their thoughtfulness towards others. Inclusion of 68 fathers was another strength, given their underrepresentation in patient-reported outcomes research [32].

Limitations included exclusion of non-English speaking families and a lack of formal screening of potential participants’ language skills, despite the importance of addressing diversity in clinical trials [33]. The participant sample was likely not representative of all families undergoing PM- those who were motivated to participate may have had more positive, or indeed less positive, experiences than the norm, which may have driven them to participate. Unfortunately, we did not have ethics board approval to further explore reasons families may have declined to participate to explore this further. Future studies should examine families’ reasons for declining to identify potential barriers to participation and strategies to improve sample representativeness. It was not possible to account for patients’ disease progression over time and the sample size was insufficient to examine differences by cancer type or treatment received. Given their poor prognosis, the patient sample size was small and was therefore insufficient to examine whether parents and children from the same family shared similar perspectives, and whether any changes in their opinions moved in the same direction throughout their participation in the trial. It would also have been valuable to further explore the experiences of patients whose parents were not able to participate in PRISM-Impact (e.g. due to a language barrier in the older generation). These are important areas for future research. As PM programmes expand and the evidence base for new therapeutic options recommended by these programmes improves, it will also be important to assess whether families’ hopes realistically reflect the potential medical benefit of PM.

Despite its limitations, this study demonstrates that parents and patients are eager to engage in PM, and provides useful guidance for improvements. Their support for PM was sustained over time and did not diminish with the child’s death. Taken together, our data demonstrate that families hold many hopes when enroling in PM, balanced by some concerns. Even when their child did not survive, few parents regretted participation, citing prolongation of hope, potential additional time with their child, reassurance of having done all that was possible for their child, and benefits for future children, as key benefits.

### Supplementary information

Supplementary Material

## Data Availability

De-identified individual participant data and the PRISM-Impact data dictionary can be made available on request to the corresponding author. Data will be available from publication of the article, with no end date.
