# Supplementary material for: Hopes, concerns, satisfaction and regret in a precision medicine trial for childhood cancer: a mixed-methods study of parent and patient perspectives
Source: Br J Cancer. 2023 Sep 19;129(10):1634–44. doi: 10.1038/s41416-023-02429-1 (PMC10645918; doi:10.1038/s41416-023-02429-1)
Supplement: Supplementary file 1 — Supplementary Material [file 41416_2023_2429_MOESM1_ESM.docx]

**
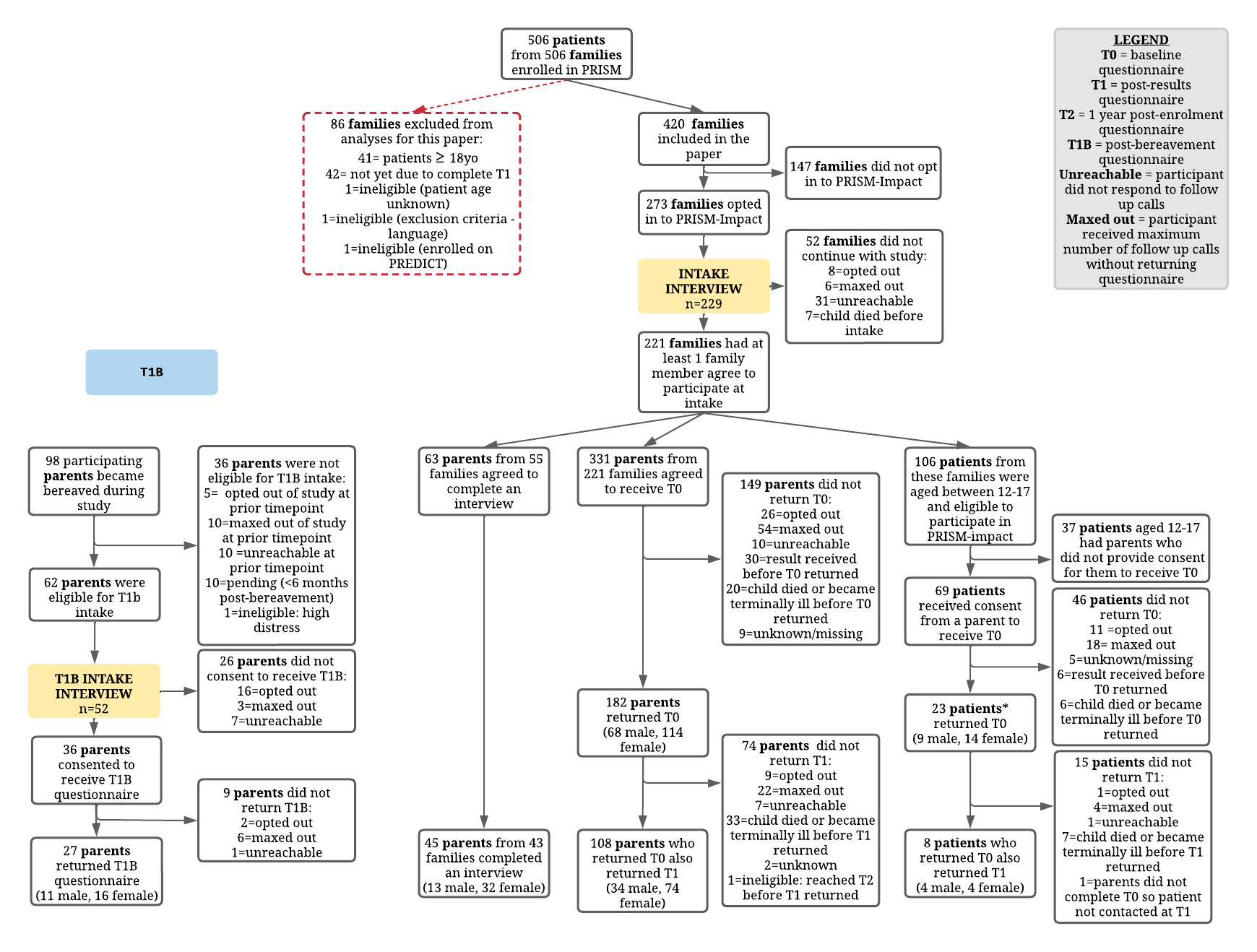
**

**Supplementary Figure 1: Participant recruitment and flow through the PRISM-Impact trial**

***** One patient completed a T0 questionnaire, despite their parents not returning their T0 questionnaires.

**NB:** 464 patients <18 years had enrolled into PRISM as at 17^th^ August 2021. Two families were ineligible for PRISM-Impact (one due to language limitations and one due to enrolment on a similar study with overlapping measures). We excluded data from 42 families who were not yet due to participate in T1, leaving 420 families eligible for PRISM-Impact, 273 of whom indicated interest in participating. 221 families had at least one parent confirm their participation in the intake call (overall family response rate=53%; 221/420). Of these, 145 families had at least one family member complete T0, resulting in a family participation rate of 66%. One hundred and eight parents from 93 families completed both T0 and T1 and 27 of the 98 parents whose child died completed T1B (28%). 152 adolescent patients were eligible for PRISM-Impact, 69 of whom had parents who consented to their participation, yielding an adolescent response rate of 45% (69/152). Twenty-three adolescents completed T0, resulting in a participation rate of 33% (23/69). Eight patients completed both T0 and T1.

Our ethics board restrictions did not enable us to document reasons for non-completion for the 76 families who did not complete a T0 questionnaire. However, of these 76 families, 11 families chose to opt-out of PRISM-Impact on follow-up, 31 families received the maximum 3 follow up calls without returning the questionnaire, and 5 families did not answer any follow-up calls from the study team, 28 families who opted in were ineligible to return T0 because they had already received their PRISM results. We had missing data for one family. Considering possible differences between participating and non-participating parents, there was no evidence to suggest that their participation differed by the child’s cancer type. However, there was some evidence to suggest that parents’ participation rates differed by hospital (p=.032). After adjusting for the child’s age, hospital and cancer type, evidence suggested that parents whose child relapsed had a higher participation rate than parents whose child had not relapsed (p=0.022).

46 adolescents did not complete their T0 questionnaire: 11 adolescents chose to opt-out of PRISM-Impact on follow-up, 18 adolescents received the maximum 3 follow up calls without returning the questionnaire, 6 became ineligible to complete T0 due to receiving their result prior to returning the questionnaire, 2 died and 4 became terminally ill. We had missing data for 5 adolescents.

Self-report data were not collected from patients aged under 12 to ensure that patients had sufficient reading levels to complete the questionnaire. While PRISM recruited patients aged 18-21 to the clinical trial, their questionnaires differed significantly from the paediatric cohort, so their experiences and preferences will be reported separately.

**Supplementary Table 1. Measures completed by parents at baseline (T0), after receipt of PRISM results (T1) and post bereavement (T1B) and data collected from the electronic medical record (eMR)**

| **Aim** | **Item** | **Measure and response option** | **T0** | **T1** | **T1B** | **eMR** |
| --- | --- | --- | --- | --- | --- | --- |
| **Demographics** | - Age  - Sex  - Whether they have other children (and their ages)  - Highest level of education  - Current employment status  - What type of health and life insurance they currently have  - Financial support  - Annual household income  - Marital status  - Cultural background  - Indigenous status  - First language  - Religious background  - Geographic location (postcode)  - Previous participation in clinical trials | Mixture of open-ended (e.g. postcode) and forced choice options (e.g. employment status) depending on the question. | **X** | **-** | **-** | **-** |
| **Clinical information of child** | - Child sex  - Child age at diagnosis  - Child age at enrolment on PRISM  - Number of relapses for child at enrolment on PRISM  - Diagnosis of child enrolled in PRISM | Data extracted from study electronic medical record for all patients of participating parents | **-** | **-** | **-** | **X** |
| **Hopes and concerns at enrolment in PRISM** | For each statement, please tick the answer that most reflects your thoughts about the PRISM study…   - I hope it will increase my child’s chance of being cured - I hope that doing this testing will provide me with peace of mind - I hope it will help find cures for future patients - I hope it will help provide information to me and the doctor about my child’s cancer - I hope it will give my child a greater number of treatment options - I worry that I may learn information about my child’s cancer that would be stressful or cause anxiety - I worry that I may learn that my child’s cancer is less treatable or more aggressive than previously thought - I worry that the information learned in this study will not be kept private - I worry that the information learned could hurt my family’s ability to get insurance - I worry that the information learned could hurt my family’s ability to get or keep a job - I worry that the results may take a long time to come back | Adapted from Marron et al. (2016)^8^. The Marron scale was administered after receipt of results, so was worded in the past tense. “Extremely true”, “Very true”, “Somewhat true”, “A little true”, “Not at all true”. | X | - | - | - |
| **Perceived impact of PRISM** | Has participation in the main PRISM study been   - burdensome to you in any way? - beneficial to you in any way? | “Not at all”, “a little”, “somewhat”, “quite a bit”, “very much” | X | X | X | - |
|  | Would you recommend being involved in the PRISM study to other parents? | “Yes”, “no”, “unsure” | X | X | X | - |
| **Satisfaction with having participated in PRISM** | At the moment, how satisfied are you with your decision about being part of PRISM? | 0 (“not at all”) to 100 (“completely”) | X | X | X | - |
|  | Thinking about your decision to participate in the PRISM study, please indicate to what extend each statement is true for you AT THIS TIME   - I am satisfied that I was adequately informed about the issues important to making my decision - The decision I made was the best decision possible for me personally at the time - I was satisfied that my decision was consistent with my personal values - I am satisfied that this was my family’s decision to make - I am satisfied with the decision I made | “Strongly disagree”, “Disagree”, “Neither agree nor disagree”, “Agree”, “Strongly agree” (Satisfaction with Decision Scale, Cronbach’s alpha =0.86)^18^ | - | X | X | - |
|  | Thinking about the decision you made about participating in the PRISM study   - It was the right decision - I regret the choice that was made - I would go for the same choice if I had to do it over again - The choice did me a lot of harm - The decision was a wise one | “Strongly disagree”, “Disagree”, “Neither agree nor disagree”, “Agree”, “Strongly agree” (Decision Regret Scale, Cronbach's alpha = 0.81 to 0.92)^17^ | - | X | X | - |

**Supplementary Table 2. Measures completed by patients at baseline (T0) and after receipt of PRISM results (T1) and data collected from the electronic medical record (eMR)**

| **Aim** | **Item** | **Measure and response option** | **T0** | **T1** | **eMR** |
| --- | --- | --- | --- | --- | --- |
| **Clinical/demographics information** | - Sex  - Age at diagnosis  - Age at enrolment on PRISM  - Number of relapses at enrolment in PRISM  - Diagnosis | Data extracted from study electronic medical record for all participating patients | **-** | **-** | **X** |
| **Hopes and concerns** | Please tell us how much you agree with these statements about being a part of the PRISM study.   - I hope it will increase my chance of being cure - I hope it will help find cures for future patients - I hope it will teach me about my cancer - I worry that being part of this study will cause me extra stress and worry - I worry that being part of this study will cause my family extra stress and worry - I worry that the results may take a long time to come back | Adapted from Marron et al. (2016)^8^. The Marron scale was administered after receipt of results, so was worded in the past tense. “Extremely true”, “Very true”, “Somewhat true”, “A little true”, “Not at all true” | X | - | - |
| **Perceived impact of PRISM** | Has being a part of the PRISM study been hard (or burdensome) in any way? | “Not at all”, “a little”, “somewhat”, “quite a bit”, “very much” | X | X | - |
|  | Has being a part of the PRISM study been good (or beneficial) in any way? |  | X | X | - |
| **Satisfaction** | At the moment, how satisfied are you with your decision about being part of PRISM? | 0 (“not at all”) to 100 (“completely”) | X | X | - |
|  | I regret being part of this study | “Extremely true”, “Very true”, “Somewhat true”, “A little true”, “Not at all true” | X | - | - |

**Supplementary Table 3. Parents’ semi-structured interview guide**

| Topic | Questions and prompts |
| --- | --- |
| Introduction | “Sometimes telling your story may bring up a range of feelings or memories. So, just before we get started I wanted to check in to see how you are feeling. On a scale of 0-10 where 0 is “None” and 10 is “Extreme” how much distress have you experienced this past week, including today? (followed by anxiety, depression, anger and need for help) [if >8, see risk management protocol at the end of the interview schedule]”  “Do you have any questions? Please let me know if you are feeling distressed or would like to stop at any point.”  “Just to get to know you a little, could you tell me briefly a little about your child and their experience with cancer?’ |
| Satisfaction with decision | “Now, in looking back at the decision you made to participate in PRISM, how you feel about your decision?”  “If you had to make the decision to participate in PRISM again, is there anything that would help you make that decision?” |
| Recommending to others | “If you were talking to another parent whose child had cancer, would you recommend being involved in the PRISM study?”  *Prompts:*  “Why?”  “Why not?” |
| Feedback on PRISM team | “Is there anything that your treatment team, or anyone involved in the PRISM study could have done better?” |

**Supplementary Table 4. Demographics of parents participating in PRISM-IMPACT**

|  | **All participating parents (N=182)** | **Interview participants (N=45)** |
| --- | --- | --- |
| **Age, years** |  |  |
| Mean (SD) | 41∙6 (7∙4) | 43∙1 (7∙7) |
| Range | 23-67 | 29-67 |
| (missing) | 4 (2∙2%) | 3 (6∙7%) |
| **Sex, no. (%)** |  |  |
| Female | 114 (62∙6%) | 32 (71∙1%) |
| Male | 68 (37∙4%) | 13 (28∙9%) |
| **Highest level of education, no. (%)** |  |  |
| High school only | 32 (17∙6%) | 5 (11∙1%) |
| Post-high school (inc. vocational training) | 150 (82∙4%) | 40 (88∙9%) |
| **Employment, no. (%)** |  |  |
| Employed: Full-time | 82 (45∙1%) | 19 (42∙2%) |
| Employed: Part-time/casual | 48 (26∙3%) | 12 (26∙6%) |
| Not employed: Actively seeking work | 5 (2∙7%) | 1 (2∙2%) |
| Not employed: Not seeking work/retired/student | 14 (7∙6%) | 6 (13∙3%) |
| Not employed: Home duties | 32 (17∙6%) | 7 (15∙6%) |
| (missing) | 1 (0∙5%) | 0 |
| **Insurance, no. (%)** |  |  |
| Private health insurance | 66 (36∙3%) | 17 (37∙8%) |
| Life insurance | 13 (7∙1%) | 3 (6∙7%) |
| Private health and life insurance | 43 (23∙6%) | 14 (31∙1%) |
| Medicare only (public healthcare) | 60 (33∙0%) | 11 (24∙4%) |
| **Receiving financial support, no. (%)** |  |  |
| No | 98 (53∙8%) | 25 (55∙6%) |
| Government/organisation | 50 (27∙5%) | 10 (22∙2%) |
| Parent, spouse or partner | 34 (18∙7%) | 10 (22∙2%) |
| **Marital status, no. (%)** |  |  |
| Never married/never de facto | 3 (1∙6%) | 0 |
| Currently married or de facto | 158 (86∙8%) | 38 (84∙4%) |
| Separated/divorced/previous de facto/widowed | 21 (11∙5%) | 7 (15∙6%) |
| **Aboriginal or Torres Strait Islander, no. (%)** | |  |
| Yes | 1 (0∙5%) | 1 (2∙2%) |
| No | 180 (98∙9%) | 44 (97∙8%) |
| (missing) | 1 (0∙5%) | 0 |
| **Cultural or language diversity, no. (%)** |  |  |
| English-speaking Western/European | 135 (74∙2%) | 35 (77∙8%) |
| English-speaking non-Western/European | 15 (8∙2%) | 3 (6∙7%) |
| Non-English-speaking | 27 (14∙8%) | 6 (13∙3%) |
| (missing) | 5 (2∙7%) | 1 (2∙2%) |
| **Religion, no. (%)** |  |  |
| No religion | 81 (44∙5%) | 20 (44∙4%) |
| Christianity | 86 (47∙3%) | 20 (44∙4%) |
| Islam | 5 (2∙7%) | 2 (4∙4%) |
| Buddhism | 2 (1∙1%%) | 0 |
| Hinduism | 4 (2.2%) | 0 |
| Judaism | 1 (0∙5%) | 0 |
| Other religion | 2 (1∙1%) | 2 (4∙4%) |
| (missing) | 1 (0∙5%) | 1 (2∙2%) |
| **Household income, no. (%)** |  |  |
| Less than $29,999 | 23 (12∙6) | 6 (13∙3%) |
| $30,000-$59,000 | 20 (11∙0%) | 7 (15∙6%) |
| $60,000-$89,000 | 34 (18∙7%) | 6 (13∙3%) |
| $90,000-$120,000 | 22 (12∙1%) | 5 (11∙1%) |
| Greater than $120,000 | 65 (35∙7%) | 19 (42∙2%) |
| Prefer not to answer | 16 (8∙8%) | 1 (2∙2%) |
| (missing) | 2 (1∙1%) | 1 (2∙2%) |
| **Rurality, no. (%)** |  |  |
| Capital city | 115 (63∙2%) | 28 (62∙2%) |
| Other metropolitan centre | 16 (8∙8%) | 4 (8∙9%) |
| Rural and remote areas | 43 (23∙6%) | 11 (24∙4%) |
| Unknown | 8 (4∙4%) | 2 (4∙4%) |
| **Number of other children, no. (%)** |  |  |
| 0 | 33 (18∙1%) | 10 (22∙2%) |
| 1 | 67 (36∙8%) | 16 (35∙6%) |
| 2-3 | 72 (39∙6%) | 17 (37∙8%) |
| 3+ | 21 (11∙5%) | 2 (4∙4%) |
| (missing) | 1 (0∙5%) | 0 |
| **Has child participated in other clinical trials since diagnosis, no. (%)** | |  |
| Yes | 45 (24∙7%) | 15 (33∙3%) |
| No | 120 (65∙9%) | 26 (57∙8%) |
| Unsure | 3 (1∙6%) | 1 (2∙2%) |
| Note: SD = standard deviation, PRISM = PRecISion Medicine for Children with Cancer, PRISM-Impact = the psychosocial sub-study running alongside the PRISM study. NB: The average age of interview participants was 43∙1 years, compared to 41∙1 for those not participating in interviews, which was not a significantly significant difference (p=0∙138). 28% of interview participants were males, compared to 41% of those not participating in interviews, which was also not statistically significant (p=0∙172). | | |
